# Antidepressant Treatment and Manic Switch in Bipolar I Disorder: A Clinical and Molecular Genetic Study

**DOI:** 10.3390/jpm12040615

**Published:** 2022-04-11

**Authors:** Chih-Ken Chen, Lawrence Shih-Hsin Wu, Ming-Chyi Huang, Chian-Jue Kuo, Andrew Tai-Ann Cheng

**Affiliations:** 1 Community Medicine Research Center & Department of Psychiatry, Chang Gung Memorial Hospital, Keelung 204, Taiwan; kenchen@cgmh.org.tw; 2 College of Medicine, Chang Gung University, Taoyuan 333, Taiwan; 3 Graduate Institute of Biomedical Sciences, China Medical University, Taichung 404, Taiwan; shwu@mail.cmu.edu.tw; 4 Taipei City Psychiatric Center, Department of General Psychiatry, Taipei City Hospital, Taipei 10341, Taiwan; mingchyihuang@gmail.com (M.-C.H.); tcpckuo@seed.net.tw (C.-J.K.); 5 Department of Psychiatry, School of Medicine, College of Medicine, Taipei Medical University, Taipei 106, Taiwan; 6 Institute of Biomedical Sciences, Academia Sinica, Taipei 11529, Taiwan

**Keywords:** bipolar I disorder, bipolar depression, antidepressant treatment, manic switch, genome-wide association study

## Abstract

Affective switch is an important clinical issue when treating bipolar disorder. Though commonly seen in clinical practice, the benefits of prescribing antidepressants for bipolar depression are still controversial. To date, there have been few genetic studies and no genome-wide association study (GWAS), focusing on manic switch following bipolar depression. This study aims to investigate the effects of individual genomics and antidepressant medication on the risk of manic switch in bipolar I disorder (BPI). A total of 1004 patients with BPI who had at least one depressive episode with complete data on antidepressant treatment and outcome were included. Clinical assessment of mania and depression was performed by trained psychiatric nurses and psychiatrists using the Chinese version of the Schedules for Clinical Assessment in Neuropsychiatry (SCAN), and the diagnosis of BPI was made according to DSM-IV criteria. Manic switch was defined as a manic episode occurring within eight weeks of remission from an acute depressive episode. The age at first depressive episode of the study patients was 30.7 years (SD 12.5) and 56% of all patients were female. GWAS was carried out in a discovery group of 746 patients, followed by replication in an independent group of 255 patients. The top SNP rs10262219 on chromosome 7 showed the strongest allelic association with manic switch (*p* = 2.21 × 10^−7^) in GWAS, which was however not significantly replicated. Antidepressant treatment significantly (odds ratio 1.7; 95% CI 1.3–2.2; *p* < 0.001) increased the risk of manic switch. In logistic regression analysis, the CC genotype of rs10262219 (odds ratio 3.0; 95% CI 1.7–5.2) and antidepressant treatment (odds ratio 2.3; 95% CI 1.4–3.7) significantly increased the risk of manic switch with a joint effect (odds ratio 5.9; 95% CI 3.7–9.4). In conclusion, antidepressant medication and rs10262219 variants jointly increased the risk of manic switch after bipolar depression.

## 1. Introduction

Bipolar disorder is characterized by episodic recurrent mania or hypomania and major depression, associated with great morbidity and mortality [1]. Bipolar depressive episodes are more pervasive with greater burden in terms of economic costs, functioning, caregiver burden, and suicide than manic episodes, and their treatments are more challenging than those in manic episodes [2]. Patients with bipolar disorder average 45% of time ill during long-term follow-up, and depression accounts for 72% of time ill [3]. In addition, patients with bipolar disorder typically require long-term pharmacological treatment to prevent recurrent episodes of depression and mania [4]. Treatment of bipolar depression remains unsatisfactory although some modern antipsychotics (particularly lurasidone, olanzapine + fluoxetine, and quetiapine) and the anticonvulsant lamotrigine are available. The value and safety of antidepressants remain controversial [5]. However, evidence-based guidelines are often not followed by prescribers, and, in some countries, the most common form of treatment is antidepressant monotherapy [6,7,8].

Manic switch is an important clinical issue when treating bipolar depression. Compared with giving a mood stabilizer alone, short-term administration of an additional antidepressant yielded neither major protection from depression nor a substantial increase in the risk of mania [9,10,11]. However, prolonged use is associated with an increased risk of treatment-emergent mania or hypomania [7,12,13]. Retrospective data obtained from patients hospitalized between 1920 and 1959 show a rate of 29% for naturalistic switching from depression to hypomania or mania [14]. The frequency of mood switching associated with acute antidepressant therapy is 27% in a trial with small samples [15]. Hypomanic/manic switches occur in 14.0~19.3% of the acute treatment trials and in 33.0~36.7% of continuation trials of adjunctive treatment with antidepressants in bipolar depression [16,17,18]. However, a naturalistic study on bipolar disorder concludes that the use of antidepressants does not influence the daily rate of switching from depression to mania [19].

So far, the progress of uncovering single nucleotide polymorphisms (SNPs) underpinning treatment efficacy in bipolar disorder is encouraging [20,21]. For example, genome-wide association study (GWAS) has identified specific SNPs associated with response to lithium treatment in bipolar disorder to be replicated [22,23,24,25]. To date, however, there have been few genetic studies [26], and no GWAS focusing on manic switch following bipolar depression has been reported. In the present report, we will first clarify whether antidepressants can increase the risk of manic episodes and examine differential risks of switching from depression to mania among various antidepressants in Taiwanese Han patients with bipolar I (BPI) disorder. Then, results of a GWAS for manic switch after bipolar depression will be presented.

## 2. Results

### 2.1. Demographic and Clinical Characteristics

**Figure 1:**
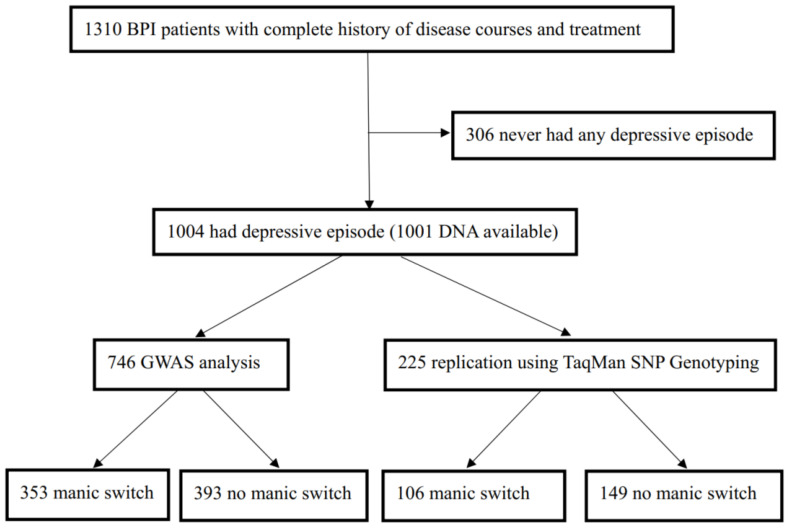
Case selection profile.

Case selection profile was showed in Figure 1, and 1004 BPI patients were selected to further analyses. Durations of antidepressant treatment ranged from 1 to 168 weeks (median: 9.0 weeks). Durations between remissions from bipolar depression to a manic switch ranged from 0 to 8 weeks (median: 3.0 weeks). Table 1a shows the demographic and clinical characteristics of the study patients. The mean age of the first depressive episode was 30.7 years old. There was no significant gender difference in rates of antidepressant treatment (44.0% in males, 56.0% in females). The overall rate of manic switch was 39.7%, with no significant gender difference (male 39.4%, female 40.0%). Following their first depressive episode, 76.2% of study patients had a subsequent manic episode. There was no significant difference in rates of a subsequent manic episode between patients with and without antidepressant treatment. However, the former were significantly (*p* < 0.001) more likely to have a manic switch after remission of depression than the latter (46.2% and 33.1%, respectively) or recurrence of mania within one year. The types of antidepressants for the first depressive episode included SSRI (Selective Serotonin Reuptake Inhibitor) (25.7%), TCA (Tricyclic Antidepressants) (9.3%), SNRI (Serotonin and Norepinephrine Reuptake Inhibitor) (6.3%), and other antidepressants (9.7%) (Table 1b). There was no significant association between rates of manic switch and types of antidepressants. Of all patients, 28.7% received lithium treatment, 40.2% received antipsychotics, and 31.6% received other mood stabilizers (including valproate 17.6%). The most commonly used antipsychotics were sulpiride (7.1%) and quetiapine (5.9%). The use of lithium, antipsychotics, or other mood stabilizers was not associated with the risk of manic switch.

**Figure 2:**
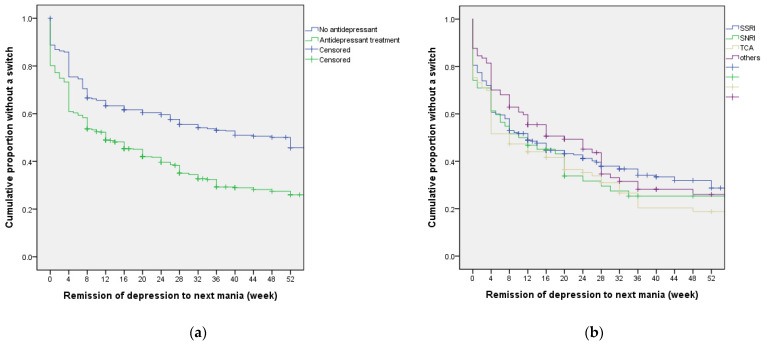
Survival functions of manic switch or recurrence after remission of the first depressive episode among bipolar I patients. (**a**) Among patients with bipolar I disorder (N = 1004), 511 received antidepressant treatments, while 493 did not for their first depressive episodes. Median survival time to manic switch or recurrence following remission of the first depressive episode was 12.0 (95% CI 8.5–15.5) weeks and 52.0 (95% CI 39.9–64.1) weeks for those with and without antidepressant treatment, respectively (chi-square = 68.2, *p* < 0.001). (**b**) Among patients who had antidepressant treatments for the first depressive episode (N = 511), the median survival time to the manic episode for those treated with SSRI (N = 257), SNRI (N = 62), TCA (N = 93), and other antidepressants (N = 99) were 12.0 (95% CI 7.9–16.1) weeks, 10.0 (95% CI 0.0–21.2) weeks, 8.0 (95% CI 4.5–11.5) weeks, and 20.0 (95% CI 7.9–32.1) weeks, respectively. Patients treated with TCA had a lower mean survival time than patients treated with other antidepressants (Generalized Wilcoxon test chi-square = 4.6, *p* = 0.032). SSRI: Selective serotonin reuptake inhibitors; SNRI: Serotonin and norepinephrine reuptake inhibitors; TCA: Tricyclic antidepressants.

Among the subjects, 156 (39.1% of those with manic switch) had a manic switch within one week after remission of bipolar depression. Figure 2a shows the survival curves of manic switch or recurrence of manic episode after remission of the first depressive episode. Patients treated with antidepressants were more likely than those without to turn into a manic switch after the first depressive episode.

Among the first depressive episodes with antidepressant treatments (N = 511), the mean survival time for those with antidepressant monotherapy and those with concomitant mood stabilizers or antipsychotics was 8.0 (95% CI 2.9–13.1) weeks and 13.0 (95% CI 8.1–17.9) weeks, respectively. There was no significant difference in survival time to the next manic episode between those with antidepressant monotherapy and those with concomitant mood stabilizers or antipsychotics (chi-square = 0.1, *p* = 0.744). Among patients whose first depressive episode was treated with antidepressants (N = 511), as shown in Figure 2b, the median survival time to the manic episode for those treated with SSRI (N = 257), SNRI (N = 62), TCA (N = 93) and other antidepressants (N = 99) were 12.0 (95% CI 7.9–16.1) weeks, 10.0 (95% CI 0.0–21.2) weeks, 8.0 (95% CI 4.5–11.5) weeks, and 20.0 (95% CI 7.9–32.1) weeks, respectively. Patients treated with TCA had a lower mean survival time than patients treated with other antidepressants (Generalized Wilcoxon test chi-square = 4.6, *p* = 0.032).

### 2.2. Genome-Wide Association Study

**Figure 3:**
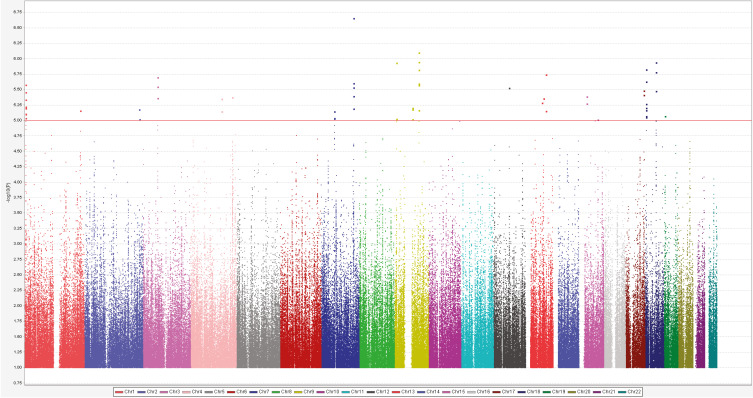
Genome-wide association between single-nucleotide polymorphisms (SNPs) and manic switch after bipolar depressive episodes. Results from the association between individual SNPs and manic switch after depressive episodes treated with antidepressants in 746 patients with bipolar I disorder. The negative log of the P value for the association, as calculated by means of the chi-square test for the dominant model, is plotted against the chromosomal location across the genome. The horizontal line indicates the significance level of 1.0 × 10^−6^, which was achieved by a few SNPs. The highest SNPs on chromosome 7 are located in the intergenic region between *LUZP6* and *CHRM2*.

The principal component analysis did not find substantial population stratification and cryptic relationship among the 746 GWAS patients (Figure S1). Manhattan plot constructed by the allelic model showed no SNP associated with manic switch after bipolar depressive episodes at genome-wide significance (Figure 3). Q-Q plot of the allelic model p values is shown in the Supplementary Materials (Figure S2). The top-ten significant SNPs are listed in the Supplementary Materials (Table S1). The Top SNP rs10262219 on chromosome 7, located in the intergenic region between leucine zipper protein 6 (*LUZP6*) and cholinergic receptor muscarinic 2 (*CHRM2*), showed the strongest allelic association with manic switch (*p* = 2.21 × 10^−7^). A few SNPs near the rs10262219 also showed suggestive association (*p* < 10^−5^) (Figure S3). The replication did not reach a significant association (*p* = 0.40), and all samples (combined GWAS and replication) showed *p* = 6.44 × 10^−7^ in the allelic model. We further analyzed the dominant model for rs10262219 and found an increased risk for manic switch with the CC genotype (odds ratio 2.5; 95% CI 1.7–3.6) (Table 2). The genotypic association for rs10262219 in the replication group did not reach statistical significance. However, logistic regression analysis showed that the CC genotype of rs10262219 (odds ratio 3.0; 95% CI 1.7–5.2) and antidepressant treatment (odds ratio 2.3; 95% CI 1.4–3.7) increased the risk of manic switch with a joint effect of odds ratio 5.9 (95% CI 3.7–9.4).

## 3. Discussion

In this retrospective cohort study, we have demonstrated that antidepressant treatment significantly increased the risk of manic switch after remission of depression. The SNP rs10262219 on chromosome 7 showed the strongest allelic association with manic switch though not reaching the level of significance after a Bonferroni correction. To our knowledge, this is the first GWAS searching for the genetic marker of manic switch following bipolar depressive episodes.

Our study found that manic switch after remission of first depressive episode was more frequently observed in patients treated with antidepressants than those without. The overall manic switch rate (39.7%) is in line with the figure reported from a multi-center study (a lifetime prevalence of 44.4%) [27]. Among the subjects with manic switch, 39.1% had a manic episode within one week after remission of the previous depressive episode. The possibility that some mixed affective episodes were classified into manic switch after bipolar depression could be ruled out. The manic switch rate in this study might be inflated in this way. Our study enrolled patients with BPI only and that could have contributed partly to the high switch rate in this study. An analysis of randomized trials of adjunctive treatment with antidepressants in bipolar depression concludes that hypomanic/manic switch rate is higher in patients with BPI (30.8%) than those with bipolar II disorder (18.6%) [18]. The manic switch rate in our study was higher than that among patients with BPI by Leverich et al. (2006), which was a prospective clinical trial with all study subjects receiving mood stabilizers under close monitoring of drug adherence [18]. Definitions of manic switch are different between these studies.

Although antidepressant-associated manic switch was reported to be more commonly observed in female than in male patients in some previous studies [28,29], no such gender difference was observed in our study. Methodological differences may account for this dissimilarity. For example, in the study by Truman et al. (2007), manic switch is defined as a report of mania, hypomania, or mixed episodes within the first 12 weeks of treatment with an antidepressant. This definition entirely excludes patients without any antidepressant treatment. We defined manic switch in this study as a manic episode occurring within eight weeks of remission from an acute depressive episode, according to the recommendations of the International Society for Bipolar Disorder (ISBD) Task Force [30].

Our finding that antidepressant treatment is associated with an increased risk of manic episode after remission of depression in bipolar patients is in line with previous studies and reviews [27,31]. In previous studies [28,32] including a meta-analysis [33], manic switch from depression was found to occur more frequently during treatment with TCA than with non-TCA drugs in bipolar inpatients. Our study revealed that TCA drugs were more commonly associated with manic switch than other antidepressants in bipolar I disorder. These findings were in line with the conclusions from the International Society for Bipolar Disorders (ISBD) Task Force Report on Antidepressant Use in Bipolar Disorders [34]. The mechanism behind the significant association between the type of antidepressants used and the risk of experiencing a manic switch needs further exploration. Two retrospective naturalistic studies separately reported that mood stabilizers as an adjunct to antidepressants in bipolar depression significantly reduced the risk of antidepressant-associated manic switch [7,35]. However, a meta-analysis on mania associated with antidepressant treatment did not find a clear-cut benefit of adjunct mood stabilizers with antidepressant treatment in the prevention of antidepressant-associated manic switch [33]. Our study found that concurrent use of mood stabilizer or antipsychotics did not reduce the risk of manic switch.

Our manic switch GWAS revealed several SNPs reaching the significance level of 1.0 × 10^−6^. The highest SNPs on chromosome 7 are located in the intergenic region between *LUZP6* and *CHRM2*. The SNP rs10262219 is located in the intergenic region between *LUZP6* and *CHRM2* genes and has no known biological function. The *CHRM2*, one of the muscarinic receptors, involves the cholinergic system and plays an important role in the pathophysiology of mood disorders [36,37]. Positron emission tomography studies have reported a decrease in the CHRM2 selective agonist [18F]FP-TZTP in the anterior cingulate cortex of individuals with bipolar disorder [36]. Decreased CHRM2 receptor binding and expression have been noted in the prefrontal cortex of the post-mortem tissue from individuals with bipolar disorder [37]. The imbalance between central cholinergic and adrenergic neurotransmitter activity may contribute to inducing mania and depression. It is suggested that a hypocholinergic-hyperadrenegic state may cause mania, whereas a hypercholinergic-hypoadrenegic state may contribute to the symptoms of depression [38]. In a rat model, the increasing levels of CHRM2 protein may be involved in the mechanisms of action of mood stabilizers and tricyclic antidepressants [39]. The role of CHRM2 in depression-mania switch phenomenon in bipolar disorder patients and the mechanisms behind warrant further investigation.

The phenotype of manic switch has raised great concerns. In many studies aiming to investigate treatment emergent switch to mania, they only include patients on antidepressants. If the aim is to analyze the naturally occurring switch to mania in bipolar disorder, then patients on antidepressants should be excluded. This study analyzed switch to mania within eight weeks after remission from bipolar depression rather than treatment emergent switch. Due to consideration of the sample size for GWAS, we did not exclude subjects on antidepressant treatment or analyze those on antidepressant treatment exclusively. Manic switch in this study may include naturally occurring switch and treatment-emergent switch.

Among patients with manic switch, 39.1% had a manic episode within one week after remission of the previous depressive episode. The possibility that some mixed affective episodes were classified into manic switch after bipolar depression could not be ruled out. The manic switch rate in this study might be inflated in this way.

There are limitations to this study. First, the sample size was relatively small, which might be responsible, at least in part, for our findings of no statistically significant association for several SNPs after correction for multiple testing. However, the frequency of risk genotype CC was higher in patients with manic switch than those without in both discovery and replication groups (Table 2). Second, our study only included patients of Han ancestry, which has the important advantage of reducing the likelihood of confounding by population structure but also limits the generalizability of our results. Third, the retrospective nature of the study indicates that our findings can only suggest an association. Fourth, there might be selection bias as treated patients in this study are more likely to have a higher severity in symptoms of depression, and those treated with antidepressants and concomitant mood stabilizers or antipsychotics might be more prone to manic episodes than their counterparts treated with antidepressant monotherapy.

In conclusion, antidepressant treatment for bipolar depressive episodes significantly increased the risk of manic switch. The top SNPs identified from the GWAS for manic switch may deserve further investigation in the future.

## 4. Materials and Methods

### 4.1. Study Patients

Study patients were selected from a total of 1807 patients with a BPI diagnosis with a complete history of disease course and treatment, recruited from 52 psychiatric departments of general hospitals and psychiatric institutions in the Taiwan Bipolar Consortium (TBC) [40] up to December 2018. The TBC aims to understand genetic vulnerability of BPI and to conduct a pharmacogenetic study of mood stabilizers. All of them were diagnosed according to DSM-IV [41] criteria for BPI disorder. Patients with other psychotic and affective disorders were excluded. For the analyses of manic switch, we first identified 1310 patients with BPI with a complete history of disease course and treatment (see case selection profile in Figure 1). Among the 1310 patients with BPI, 1004 had had at least one major depressive episode and were included in the analyses of manic switch. The study was approved by the institutional review board of all the participating hospitals and Academia Sinica, Taiwan. All the study subjects provided a written informed consent.

### 4.2. Assessments

Clinical assessment of mania and depression was performed by trained psychiatric nurses and psychiatrists using the Chinese version of the Schedules for Clinical Assessment in Neuropsychiatry (SCAN) [42], supplemented by available medical records and reports from key family members and in-charge psychiatrists. The assessment of affective switch and antidepressant treatment in BPI disorder was based on a life chart with graphical presentation of lifetime clinical course prepared for every patient recruited by the TBC. This life chart included all manic, hypomanic, and depressive episodes with onset year and month, duration, and severity, all doses of and duration of treatment with psychotropic drugs and mood stabilizers ever prescribed, drug adherence recorded in medical charts during treatment at outpatient clinics, all recorded blood levels of mood stabilizers, and any adverse drug reactions. Mixed episodes were categorized into manic episodes in this study. This graphical life chart was presented on the basis of integrated information gathered from direct interviews with patients and their key family members, interviews with in-charge psychiatrists, and a thorough medical chart review.

### 4.3. Antidepressant Treatment and Affective Switch

We then compared frequencies of affective switches from depression to mania between those with and without antidepressant treatments. The first documented depressive episode was selected as the index episode. Antidepressant treatments with doses less than 1/2 defined daily dose recommended by the WHO Collaborating Center for Drug Statistics Methodology and with a duration of less than two weeks were included in the “no antidepressant treatment” group unless these treatments were apparently associated with a manic switch. Affective switches or other courses and outcomes in bipolar disorder were defined according to the recommendations of the International Society for Bipolar Disorder Task Force [30]. A manic switch and a manic recurrence were defined as a manic episode occurring within and >8 weeks, respectively, of remission from an acute depressive episode.

### 4.4. Genome-Wide Association Study (GWAS)

Genotyping was performed using the Illumina HumanOmni1-Quad BeadChip (N = 936) and the HumanOmni2.5-Quad BeadChip (N = 575) by Chun-Tai Co. (Taipei, Taiwan). For this study, we integrated the two genome-wide SNP data sets through imputation with 1000 Genomes. To bridge the two sets, we also genotyped 82 of the first 936 patients using the HumanOmni2.5-Quad BeadChip. The two gene chips shared about 750 K common SNPs, thus we could check the genotyping consistency of the two sets. Genotype calling for the two data sets was determined by Beadstudio (Illumina) using default parameters. The genotype imputation method, IMPUTE2 [43,44,45], was performed under default setting to estimate the genotypes of SNPs not on array. In the imputation process, reference haplotypes were curated from 1000 Genomes Project Phase III [46]. To improve the efficiency, we performed whole genome imputation in every 5 Mb chunk, respectively. ANNOVAR [47] was used to annotate the functional consequences of single nucleotide variants found in our dataset. The imputation dataset included genotyping information from 1429 subjects of the TBC. Among the 1004 patients, 746 with genotyping data were included in the GWAS for affective switch. The following quality control of the genotype data was implemented for each data set to exclude SNPs and individuals before the imputation: (1) Individuals with a call rate <98%, (2) *p* < 1.0 × 10^−4^ for Hardy-Weinberg violation, (3) SNPs with MAF <5%, (4) samples with first-degree cryptic relationships, (5) samples that were potentially contaminated. The imputation dataset included genotyping information from 1429 subjects of the Taiwan Bipolar Consortium. Among the 1004 patients, 746 with genotyping data were included in the GWAS for this study.

Except for 3 patients with no DNA available, the remaining 255 of the 1004 subjects without genotyping information were used for replication. We used the TaqMan SNP Genotyping Assays (ABI: Applied Biosystems Inc. Foster City, CA, USA) for replication. The primers and probes of the replicated SNP(s) were from an ABI assay on demand (AOD) kit. Reactions were carried out according to the manufacturer’s protocol (TaqMan SNP Genotyping Assays, protocol, Part Number 4,332,856 Rev. C). The probe fluorescence signal detection was performed using an ABI Prism 7900 Real-Time PCR System.

### 4.5. Statistical Analysis

Variables were presented as either mean (± standard deviation) or frequency (%). Chi-square test or t test was used to compare the differences between depressive episodes with or without affective switch for categorical or continuous variables, respectively. Logistic regression was used to analyze multivariate risk factors of affective switches. Survival curves of affective switches from depressive to manic episodes were analyzed using Kaplan-Meier cumulative survival analyses. The statistical software package IBM SPSS Statistics ver. 21.0 (IBM Co., Armonk, NY, USA) was utilized for statistical analyses. All tests were two-tailed, and *p* values < 0.05 were considered significant.

Principal component analysis for all study subjects based on the genome-wide IBS (identical by state) pairwise distances was performed using PLINK v. 1.9 (https://www.cog-genomics.org/plink2, accessed on 10 March 2022) [48]. GWAS was carried out for the discovery groups by comparing allele/genotype frequencies between patients with BPI with and without a history of manic switch. The threshold P value was set at 1.05 × 10^−8^ after a Bonferroni correction for the number of SNPs (4,750,978). When no SNP reached the significance level after Bonferroni correction, top SNP was considered for further examinations. Quantile-quantile (Q-Q) plots were then used to examine P-value distributions. The calculation was performed using PLINK v. 1.9 [48]. Top SNP identified in the GWAS were replicated in the replication group. The χ^2^ test for replication and evaluation of diagnostic tests were performed by SAS 9.4 (SAS Institute Inc., Cary, NC, USA).

## Figures and Tables

**Table 1:** Demographic and clinical characteristics, comparing statistics of study patients with bipolar I disorder.

**(a) Antidepressant Treatment**				
	Total	Treatment with antidepressants		
	N = 1004	No (N = 493)	Yes (N = 511)	*p* Value
Female gender, N (%)	562 (56.0)	262 (53.1)	300 (58.7)	0.086
Age at first depressive episode, mean (SD)	30.7 (12.5)	29.7 (12.0)	31.6 (12.8)	0.017
Subsequent manic episode, N (%)	765 (76.2)	384 (77.9)	381 (74.6)	0.215
Manic switch or recurrence within 1 year, N (%)	603 (60.1)	259 (52.5)	344 (67.3)	<0.001
Manic switch, N (%)	399 (39.7)	163 (33.1)	236 (46.2)	<0.001
**(b) Manic Switch**				
		Manic switch		
		No (N = 605)	Yes (N = 399)	
Female gender, N (%)	562 (56.0)	336 (55.5)	226 (56.6)	0.730
Age at first depressive episode, mean (SD)	30.7 (12.5)	30.9 (12.2)	30.4 (12.9)	0.017
Types of antidepressants				
SSRI	258 (25.7)	138 (22.8)	120 (30.1)	0.244
TCA	93 (9.3)	44 (7.3)	49 (12.3)	
SNRI	63 (6.3)	33 (5.5)	30 (7.5)	
Other antidepressants	97 (9.7)	60 (9.9)	37 (9.3)	
Lithium, N (%)	270 (28.7)	163 (28.6)	107 (28.7)	0.990
Other mood stabilizers, N (%)	317 (31.6)	185 (30.6)	132 (33.1)	0.403
Antipsychotics, N (%)	404 (40.2)	249 (41.2)	155 (38.8)	0.465

SD: standard deviation; SSRI: selective serotonin reuptake inhibitors; TCA: tricyclic antidepressants; SNRI: serotonin and norepinephrine reuptake inhibitors.

**Table 2:** Effects of antidepressants treatment and rs10262219 genotypes on manic switch after bipolar depression.

	Switch (+)	Switch (−)	Odds Ratio (95% CI)	*p* Value
**GWAS cohort, N = 746(%)**				
CC	301 (85.3%)	275 (70.0%)	2.5 (1.7–3.6)	6.6 × 10^−7^
TT + TC	52 (14.7%)	118 (30.0%)		
**Replication cohort, N = 255(%)**				
CC	77 (72.6%)	100 (67.1%)	1.3 (0.8–2.2)	0.36
TT + TC	29 (27.4%)	49 (32.9%)		
**Combined, N = 1001(%)**				
CC	378 (82.4%)	375 (69.2%)	2.1 (1.5–2.8)	1.96 × 10^−6^
TT + TC	81 (17.6%)	167(30.8%)		
Antidepressants X rs10262219 *				
Antidepressants(−) × CT + TT (ref.)			1	
Antidepressants(+) × CC			5.9 (3.7–9.4)	<10^−6^
Antidepressants(+) × CT + TT			2.3 (1.4–3.7)	7.0 × 10^−4^
Antidepressants(−) × CC			3.0 (1.7–5.2)	1.2 × 10^−4^

Ref. reference group * Joint effect of antidepressant medication and rs10262219 genotypes from logistic regression analysis.

## Data Availability

The datasets generated during and/or analyzed during the current study are not publicly available due to the limitation of study consent documents with repository deposition but are available from the corresponding author upon reasonable request.

## Supplementary Materials

The following supporting information can be downloaded at: https://www.mdpi.com/article/10.3390/jpm12040615/s1. Figure S1. The principal component analysis plot of the 1306 GWAS samples, Figure S2. Q-Q plot of the fisher’s test for allelic model p values of GWAS cohort (N = 746), Figure S3. Regional plots of genetic variants in genomic location encompass rs10262219 (±10 kbp) with genotyping data and association *p* values (−log_10_p). Table S1. Top-ten significant SNPs in GWAS at allelic model.
