# Supplementary material for: Antidepressant Treatment and Manic Switch in Bipolar I Disorder: A Clinical and Molecular Genetic Study
Source: J Pers Med. 2022 Apr 11;12(4):615. doi: 10.3390/jpm12040615 (PMC9033004; doi:10.3390/jpm12040615)
Supplement: Supplementary file 1 [file jpm-12-00615-s001.zip › jpm-1654531-supplementary.pdf]

Supplementary Materials

Chen et al. “Antidepressant treatment and manic switch in bipolar I disorder: a clinical and molecular genetic study”

Figure S1. The principal component analysis plot of the 1306 GWAS samples

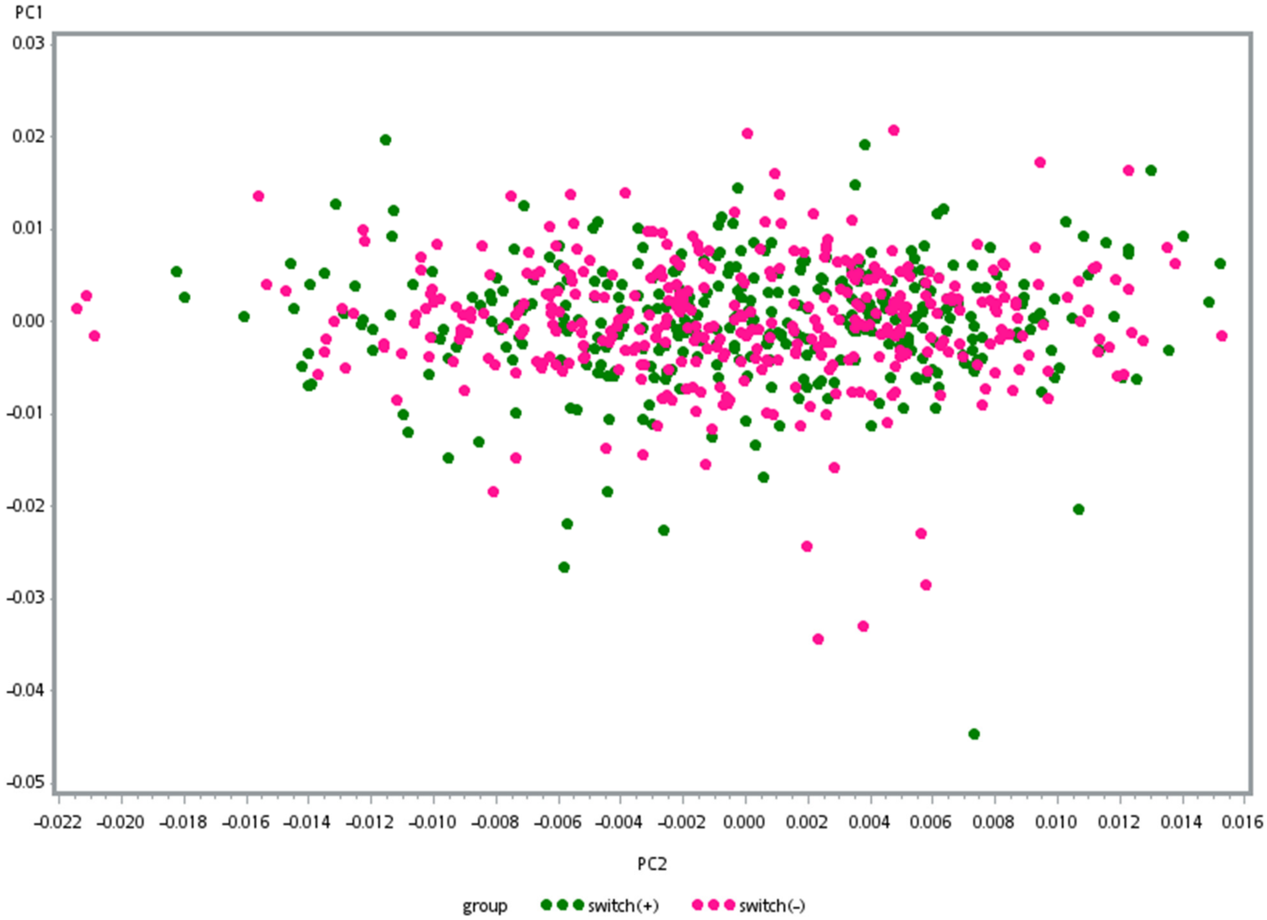

The Y and X axes are the first and second dimensions from principal component analysis based on the genome-wide IBS pairwise distances among the 746 GWAS subjects. Green crosses represented mania switch (+) and pink for mania switch (-). The two axes correspond to a reduced representation of 10,000 randomly selected SNPs into two dimensions. No clustering pattern was found and indicated that neither substantial population stratification nor cryptic relationship among the 746 subjects was found.

Figure S2. Q-Q plot of the fisher's test for allelic model p values of GWAS cohort (N=746)

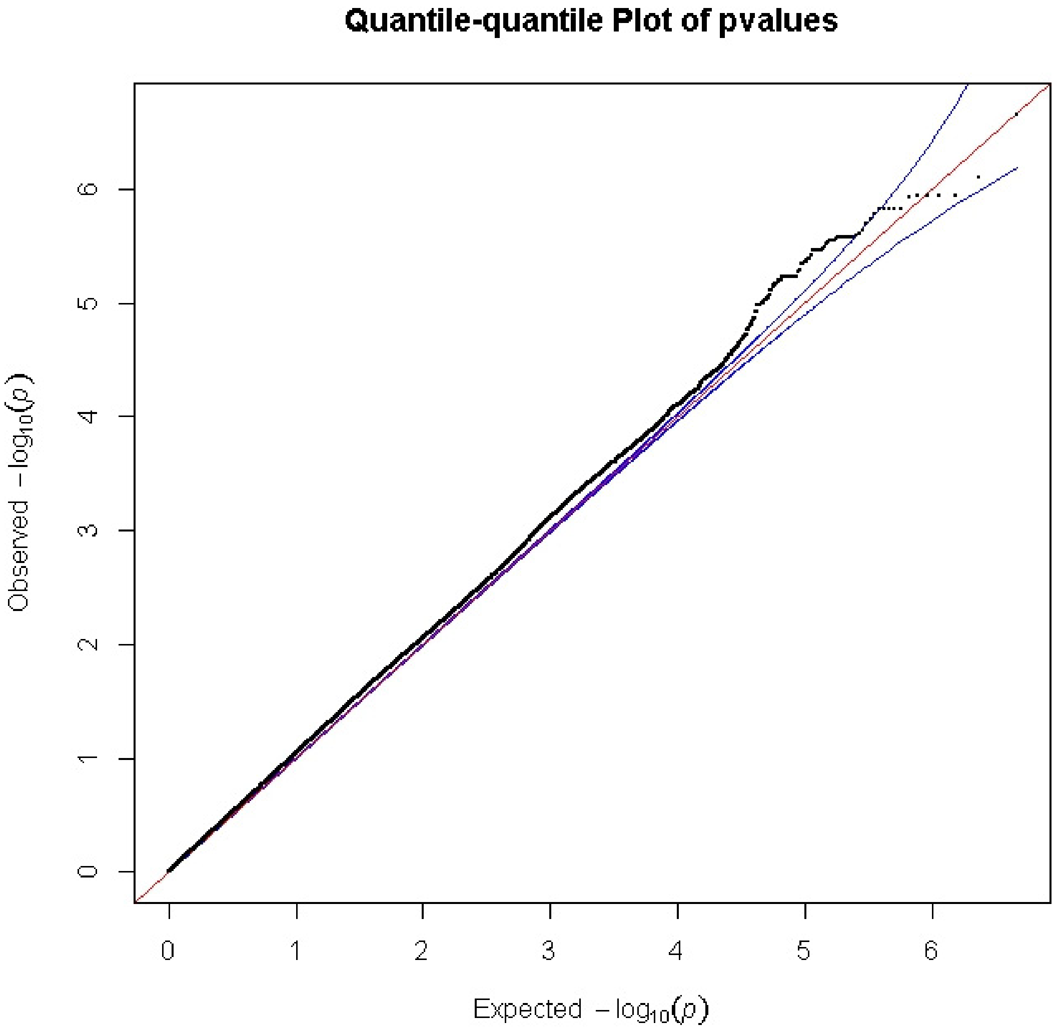

Figure S3. Regional plots of genetic variants in genomic location encompass rs10262219 (± 10kbp) with genotyping data and association P values (-log10P).

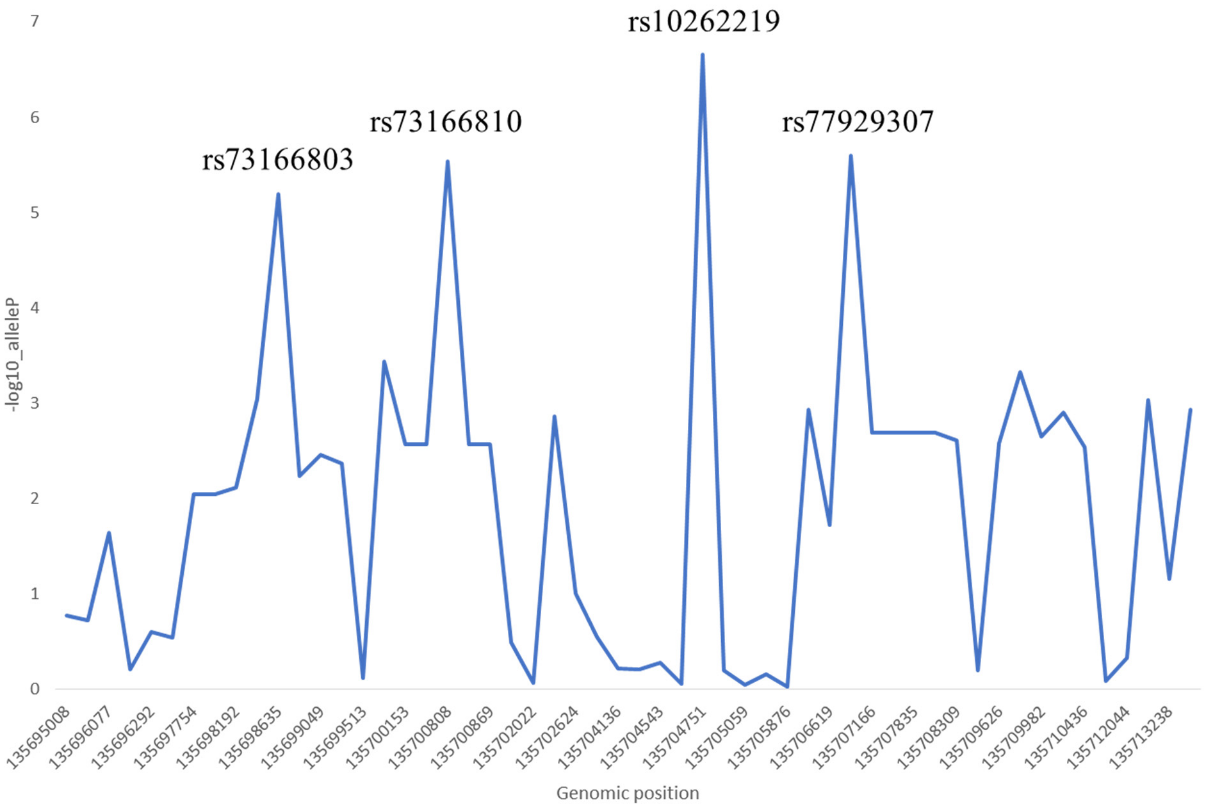

Table S1. Top-ten significant SNPs in GWAS at allelic model

SNPs	Nearest gene (s)	feature	Alleles Minor/Major	Count switch	Count None switch	P value
rs10262219	LUZP6,CHRM2*	intergenic	C/T	57/649	134/652	2.21E-07
rs1889266	COL15A1	intronic	C/G	133/573	78/708	8.06E-07
rs10116572	GALNT12,COL15A1	intergenic	C/T	132/574	78/708	1.14E-06
rs10123404	GALNT12,COL15A1	intergenic	G/C	132/574	78/708	1.14E-06
rs10988270	COL15A1	upstream	A/T	132/574	78/708	1.14E-06
rs78553128	ZBTB7C,CTIF	intergenic	C/A	398/308	344/442	1.16E-06
rs911767	TMEM261,PTPRD	intergenic	G/A	388/318	333/453	1.18E-06
rs72871106	DLGAP1	intronic	C/T	61/645	134/652	1.50E-06
rs72871111	DLGAP1	intronic	A/G	61/645	134/652	1.50E-06
rs4511627	DLGAP1	intronic	T/A	61/645	134/652	1.50E-06

*Only CHRM2 in the genes listed in the table is related to mood swings (please see discussion section). Therefore, we only verify this SNP.
